# Genetic evaluation for three-way crossbreeding

**DOI:** 10.1186/s12711-015-0177-6

**Published:** 2015-12-22

**Authors:** Ole F. Christensen, Andres Legarra, Mogens S. Lund, Guosheng Su

**Affiliations:** Department of Molecular Biology and Genetics, Center for Quantitative Genetics and Genomics, Aarhus University, Blichers Allé 20, P.O. BOX 50, 8830 Tjele, Denmark; INRA, UMR 1388 GenPhySE, BP52627, 31326 Castanet Tolosan, France

## Abstract

**Background:**

Commercial pig producers generally use a terminal crossbreeding system with three breeds. Many pig breeding organisations have started to use genomic selection for which genetic evaluation is often done by applying single-step methods for which the pedigree-based additive genetic relationship matrix is replaced by a combined relationship matrix based on both marker genotypes and pedigree. Genomic selection is implemented for purebreds, but it also offers opportunities for incorporating information from crossbreds and selecting for crossbred performance. However, models for genetic evaluation for the three-way crossbreeding system have not been developed.

**Results:**

Four-variate models for three-way terminal crossbreeding are presented in which the first three variables contain the records for the three pure breeds and the fourth variable contains the records for the three-way crossbreds. For purebred animals, the models provide breeding values for both purebred and crossbred performances. Heterogeneity of genetic architecture between breeds and genotype by environment interactions are modelled through genetic correlations between these breeding values. Specification of the additive genetic relationships is essential for these models and can be defined either within populations or across populations. Based on these two types of additive genetic relationships, both pedigree-based, marker-based and combined relationships based on both pedigree and marker information are presented. All these models for three-way crossbreeding can be formulated using Kronecker matrix products and therefore fitted using Henderson’s mixed model equations and standard animal breeding software.

**Conclusions:**

Models for genetic evaluation in the three-way crossbreeding system are presented. They provide estimated breeding values for both purebred and crossbred performances, and can use pedigree-based or marker-based relationships, or combined relationships based on both pedigree and marker information. This provides a framework that allows information from three-way crossbred animals to be incorporated into a genetic evaluation system.

## Background

Commercial pig producers generally use a terminal crossbreeding system with three breeds. In this system, F1 sows from two maternal breeds are mated to purebred boars from a breed that has high-level production traits (growth, leanness, feed efficiency) to produce pigs for slaughter. Commonly, boar lines in Europe are Duroc and Pietrain and sows are crosses between Large White and Landrace. Genetic evaluation is usually done within each of these breeds based on recorded phenotypes on purebred animals. However, ideally genetic evaluation of purebreds should incorporate phenotypes of interest recorded on crossbreds, and breeding values for performance in the three-way cross should be estimated.

Many pig breeding organisations have started to use genomic selection [1], for which genetic evaluation is often done by applying single-step methods [2–4] to handle the fact that only a fraction of the animals are genotyped. Here, the pedigree-based additive genetic relationship matrix is replaced by a combined relationship matrix based on both marker genotypes and pedigree. Genomic selection is implemented for purebreds, but it also offers opportunities for incorporating information from crossbreds and selecting for crossbred performance [5–7].

**Table 1:** Example pedigree

id	Father	Mother	Population
1	0	0	$\mathcal {A}$
2	0	0	$\mathcal {A}$
3	0	0	$\mathcal {B}$
4	0	0	$\mathcal {C}$
5	1	2	$\mathcal {A}$
6	3	5	$\mathcal {A}\mathcal {B}$
7	4	6	$\mathcal {C}(\mathcal {A}\mathcal {B})$
8	4	6	$\mathcal {C}(\mathcal {A}\mathcal {B})$

For two-way terminal crossbreeding (two breeds named $\mathcal {A}$ and $\mathcal {B}$, and all crossbred animals $\mathcal {A}\mathcal {B}$ have known purebred parents), Wei and van der Werf [8] proposed the following trivariate model:

1 $$\begin{aligned} \mathbf {y}_{\mathcal {A}}&= \mathbf {X}_{\mathcal {A}} \varvec{\beta }_{\mathcal {A}} + \mathbf {Z}_{\mathcal {A}} \mathbf {a}_{\mathcal {A}} + \mathbf {e}_{\mathcal {A}} , \nonumber \\ \mathbf {y}_{\mathcal {B}}&= \mathbf {X}_{\mathcal {B}} \varvec{\beta }_{\mathcal {B}} + \mathbf {Z}_{\mathcal {B}}\mathbf {a}_{\mathcal {B}} + \mathbf {e}_{\mathcal {B}} , \nonumber \\ \mathbf {y}_{\mathcal {A}\mathcal {B}}&= \mathbf {X}_{\mathcal {A}\mathcal {B}} \varvec{\beta }_{\mathcal {A}\mathcal {B}} + \mathbf {g}_{\mathcal {A}\mathcal {B}} + \mathbf {e}_{\mathcal {A}\mathcal {B}} , \end{aligned}$$

where the vectors $\mathbf {y}_{\mathcal {A}}$, $\mathbf {y}_{\mathcal {B}}$ and $\mathbf {y}_{\mathcal {A}\mathcal {B}}$ contain phenotypes on animals from breeds $\mathcal {A}$ and $\mathcal {B}$ and from the cross $\mathcal {A}\mathcal {B}$, respectively, and for the three populations $\mathcal {A}$, $\mathcal {B}$ and $\mathcal {A}\mathcal {B}$, the vectors $\varvec{\beta }_{\mathcal {A}}$, $\varvec{\beta }_{\mathcal {B}}$ and $\varvec{\beta }_{\mathcal {A}\mathcal {B}}$ contain fixed effects (note that intercepts should always be included!), and $\mathbf {e}_{\mathcal {A}} \sim N(\mathbf {0}, \sigma ^2_{e,\mathcal {A}} \mathbf {I})$, $\mathbf {e}_{\mathcal {B}} \sim N(\mathbf {0}, \sigma ^2_{e,\mathcal {B}} \mathbf {I})$ and $\mathbf {e}_{\mathcal {A}\mathcal {B}} \sim N(\mathbf {0}, \sigma ^2_{e,\mathcal {A}\mathcal {B}} \mathbf {I})$ are the residual error vectors. The vectors $\mathbf {a}_{\mathcal {A}}$ and $\mathbf {a}_{\mathcal {B}}$ contain breeding values for purebred performance (mating within breed) for breeds $\mathcal {A}$ and $\mathcal {B}$, respectively, and the vector of genetic values on the crossbreds, $\mathbf {g}_{\mathcal {A}\mathcal {B}}$, is related to the vectors of breeding values on purebred animals for crossbred performance (mating with the other breed), $\mathbf {g}_{\mathcal {A}}$ and $\mathbf {g}_{\mathcal {B}}$, by additive pedigree-based relationships (throughout this paper, additive genetic effects for purebred performance and for crossbred performance are denoted by $\mathbf {a}$ and $\mathbf {g}$, respectively). Each animal has then two breeding values (one related to mating within breed, e.g. $\mathbf {a}_{\mathcal {A}}$, and another related to mating to another breed to produce the cross, e.g. $\mathbf {g}_{\mathcal {A}}$) and these are correlated. Genetic correlations less than 1 are due to the presence of non-additive gene action in combination with different allele frequencies in the two breeds [9, 10], but also to genotype by environment interactions. The model also assumes different genetic variances in the two pure breeds, which is often the case in practice. Christensen et al. [11] reformulated the model using partial relationship matrices (see below) and constructed those from a combination of marker genotypes and pedigree in such a way that it could be fitted by using standard animal breeding software, i.e. a single-step method was developed.

**Table 2:** Breed $\mathcal {A}$ specific partial relationship matrix $\mathbf {A}^{\mathcal {A}}$ for the pedigree in Table 1

id	1	2	5	6	7	8
1	1	0	$\frac{1}{2}$	$\frac{1}{4}$	$\frac{1}{8}$	$\frac{1}{8}$
2		1	$\frac{1}{2}$	$\frac{1}{4}$	$\frac{1}{8}$	$\frac{1}{8}$
5			1	$\frac{1}{2}$	$\frac{1}{4}$	$\frac{1}{4}$
6				$\frac{1}{2}$	$\frac{1}{4}$	$\frac{1}{4}$
7					$\frac{1}{4}$	$\frac{1}{8}$
8						$\frac{1}{4}$

The aim of this work was to develop models for three-way terminal crosses that handle both pedigree-based and marker-based relationships, as well as combined relationship matrices based on both pedigree and marker genotypes. As indicated above, an essential part of the model is the specification of relationships such that the model can be fitted by using standard animal breeding software.

## Methods

We present a specific scenario with records on all three pure breeds and on three-way production pigs, but not on two-way crossbred sows, having in mind production traits such as daily gain, leanness or feed efficiency. However, since we will specify relationships across all five populations, it is straightforward to generalise to other scenarios with records.

**Table 3:** Breed $\mathcal {B}$ specific partial relationship matrix $\mathbf {A}^{\mathcal {B}}$ for the pedigree in Table 1

id	3	6	7	8
3	1	$\frac{1}{2}$	$\frac{1}{4}$	$\frac{1}{4}$
6		$\frac{1}{2}$	$\frac{1}{4}$	$\frac{1}{4}$
7			$\frac{1}{4}$	$\frac{1}{8}$
8				$\frac{1}{4}$

The model for this three-way terminal crossbreeding system is in principle a straightforward generalisation of the Wei and van der Werf model [8] to the following four-variate model:

2 $$\begin{aligned} \mathbf {y}_{\mathcal {A}}&= \mathbf {X}_{\mathcal {A}} \varvec{\beta }_{\mathcal {A}} + \mathbf {Z}_{\mathcal {A}} \mathbf {a}_{\mathcal {A}} + \mathbf {e}_{\mathcal {A}} , \nonumber \\ \mathbf {y}_{\mathcal {B}}&= \mathbf {X}_{\mathcal {B}} \varvec{\beta }_{\mathcal {B}} + \mathbf {Z}_{\mathcal {B}}\mathbf {a}_{\mathcal {B}} + \mathbf {e}_{\mathcal {B}} , \nonumber \\ \mathbf {y}_{\mathcal {C}}&= \mathbf {X}_{\mathcal {C}} \varvec{\beta }_{\mathcal {C}} + \mathbf {Z}_{\mathcal {C}}\mathbf {a}_{\mathcal {C}} + \mathbf {e}_{\mathcal {C}} , \nonumber \\ \mathbf {y}_{\mathcal {C}(\mathcal {A}\mathcal {B})}&= \mathbf {X}_{\mathcal {C}(\mathcal {A}\mathcal {B})} \varvec{\beta }_{\mathcal {C}(\mathcal {A}\mathcal {B})} + \mathbf {g}_{\mathcal {C}(\mathcal {A}\mathcal {B})} + \mathbf {e}_{\mathcal {C}(\mathcal {A}\mathcal {B})} , \end{aligned}$$

where notation is defined as for Eq. (1), and it is assumed that all $\mathcal {C}(\mathcal {A}\mathcal {B})$ animals have a purebred $\mathcal {C}$ father and crossbred $\mathcal {A}\mathcal {B}$ mother, and that these $\mathcal {A}\mathcal {B}$ animals all have purebred parents. Breed $\mathcal {C}$ animals have two breeding values that are correlated, $\mathbf {a}_{\mathcal {C}}$ for purebred performance (mating within breed) and $\mathbf {g}_{\mathcal {C}}$ for $\mathcal {C}(\mathcal {A}\mathcal {B})$ crossbred performance (mating between a male and a $\mathcal {A}\mathcal {B}$ crossbred female). Breed $\mathcal {A}$ animals also have two breeding values, $\mathbf {a}_{\mathcal {A}}$ for purebred performance (mating within breed) and $\mathbf {g}_{\mathcal {A}}$ for crossbred $\mathcal {C}(\mathcal {A}\mathcal {B})$ performance (mating with a breed $\mathcal {B}$ animal whose female $\mathcal {A}\mathcal {B}$ crossbred offspring is mated with a breed $\mathcal {C}$ male). Finally, breed $\mathcal {B}$ animals have two breeding values $\mathbf {a}_{\mathcal {B}}$ and $\mathbf {g}_{\mathcal {B}}$, defined similarly to the breeding values for breed $\mathcal {A}$. For each breed, association between breeding values for purebred and crossbred performances is determined by a $2\times 2$ genetic variance-covariance matrix. An essential part of the model is the specification of the additive relationships between genetic values for crossbred performance on crossbred animals and purebred animals, and in particular marker-based versions of these relationships such that pedigree-based and marker-based relationships are consistent. These relationships should also be specified in such a way that the model can be formulated using Kronecker products, allowing the model to be fitted by using Henderson’s mixed model equations and standard animal breeding software. Additive relationships are relationships between gene substitution effects and these can be defined either within populations or across populations [12]. These two approaches will be called “partial genetic” and “common genetic” approaches in the following.

**Table 4:** Breed $\mathcal {C}$ specific partial relationship matrix $\mathbf {A}^{\mathcal {C}}$ for the pedigree in Table 1

id	4	7	8
4	1	$\frac{1}{2}$	$\frac{1}{2}$
7		$\frac{1}{2}$	$\frac{1}{4}$
8			$\frac{1}{2}$

Lo et al. [13] derived the following recursive formulas for the variance and covariance of genotypic values for animals composed of multiple breeds under an additive model. Let the genotypic value of individual *i* be $g_i$, then the additive variance is:

3 $$\begin{aligned} {\text {Var}}(g_i)&= \sum _b f^b_i \sigma ^2_{g,b} + {\text {Cov}}(g_{f(i)},g_{m(i)}) \nonumber \\ & \quad + 2 \sum _b\sum _{b^{\prime }>b} (f^b_{f(i)}f^{b^{\prime }}_{f(i)}+f^b_{m(i)}f^{b^{\prime }}_{m(i)})\sigma ^2_{g,b,b^{\prime }} \end{aligned}$$

where *b* and $b^{\prime }$ denote breeds, $f^b_i$ is the breed *b* content of individual *i*, $\sigma ^2_{g,b}$ is the breed *b* genetic variance, $g_{f(i)}$ and $g_{m(i)}$ are the additive genetic values of parents *f*(*i*) and *m*(*i*), respectively, and $\sigma ^2_{g,b,b^{\prime }}$ is the breed *b* and breed $b^{\prime }$ segregation genetic variance. The additive covariance between genotypic values of individuals *i* and $i^{\prime }$ is:

4 $$\begin{aligned} {\text {Cov}}(g_i, g_{i^{\prime }}) =({\text {Cov}}(g_{f(i)},g_{i^{\prime }}) + {\text {Cov}}(g_{m(i)},g_{i^{\prime }}))/2 , \end{aligned}$$

when $i^{\prime }\ne i$ is not a descendant of *i*.

**Table 5:** Breed $\mathcal {A}\mathcal {B}$ segregation partial relationship matrix $\mathbf {A}^{\mathcal {A}\mathcal {B}}$ for the pedigree in Table 1

id	9	10
9	$\frac{1}{2}$	0
10		$\frac{1}{2}$

García-Cortés and Toro [14] showed that Eqs. (3) and (4) could be expressed as (using matrix notation):

$$\begin{aligned} {\text {Var}}(\mathbf {g})= \sum _b \sigma ^2_b\mathbf {A}^b + \sum _b\sum _{b^{\prime }>b} \sigma ^2_{b,b^{\prime }}\mathbf {A}^{b,b^{\prime }} \end{aligned}$$

where the $\mathbf {A}^b$ and $\mathbf {A}^{b,b^{\prime }}$ matrices are separately defined using recursions, and that this provides a partition of the vector of genotypic values into:

$$\begin{aligned} \mathbf {g}= \sum _b \mathbf {g}^b + \sum _b\sum _{b^{\prime }>b} \mathbf {g}^{b,b^{\prime }} , \end{aligned}$$

where all the $\mathbf {g}^b$, $\mathbf {g}^{b,b^{\prime }}$ vectors are independent, ${\text {Var}}(\mathbf {g}^b)=\sigma ^2_b\mathbf {A}^b$ and ${\text {Var}}(\mathbf {g}^{b,b^{\prime }})= \sigma ^2_{b,b^{\prime }}\mathbf {A}^{b,b^{\prime }}$. They termed matrix $\mathbf {A}^b$ as the breed *b* specific partial relationship matrix and matrix $\mathbf {A}^{b,b^{\prime }}$ the breed *b* and breed $b^{\prime }$ segregation partial relationship matrix. The vectors $\mathbf {g}^b$ and $\mathbf {g}^{b,b^{\prime }}$ depend on genetic origin, such that $\mathbf {g}^b$ is the breed *b* specific partial genetic vector, and $\mathbf {g}^{b,b^{\prime }}$ is the breed *b* and breed $b^{\prime }$ segregation partial genetic vector. Matrices $\mathbf {A}^b$ and $\mathbf {A}^{b,b^{\prime }}$ have sparse inverses that can be computed using the usual methods for the additive relationship matrix (see [14]). In this paper, the approach using a partition of the genetic effects into independent terms is named partial genetic approach.

**Table 6:** Common relationship matrix $\mathbf {A}(\varvec{\Gamma })$ for the pedigree in Table 1

id	1	2	3	4	5	6	7	8
1	$1+\frac{\gamma _{\mathcal {A}}}{2}$	$\gamma _{\mathcal {A}}$	$\gamma _{\mathcal {A}\mathcal {B}}$	$\gamma _{\mathcal {A}\mathcal {C}}$	$\frac{1}{2}+\frac{3\gamma _{\mathcal {A}}}{4}$	$\frac{1}{4}+\frac{3\gamma _{\mathcal {A}}}{8}+\frac{\gamma _{\mathcal {A}\mathcal {B}}}{2}$	$\frac{1}{8}+\frac{3\gamma _{\mathcal {A}}}{16}+\frac{\gamma _{\mathcal {A}\mathcal {B}}}{4}+\frac{\gamma _{\mathcal {A}\mathcal {C}}}{2}$	$\frac{1}{8}+\frac{3\gamma _{\mathcal {A}}}{16}+\frac{\gamma _{\mathcal {A}\mathcal {B}}}{4}+\frac{\gamma _{\mathcal {A}\mathcal {C}}}{2}$
2		$1+\frac{\gamma _{\mathcal {A}}}{2}$	$\gamma _{\mathcal {A}\mathcal {B}}$	$\gamma _{\mathcal {A}\mathcal {C}}$	$\frac{1}{2}+\frac{3\gamma _{\mathcal {A}}}{4}$	$\frac{1}{4}+\frac{3\gamma _{\mathcal {A}}}{8}+\frac{\gamma _{\mathcal {A}\mathcal {B}}}{2}$	$\frac{1}{8}+\frac{3\gamma _{\mathcal {A}}}{16}+\frac{\gamma _{\mathcal {A}\mathcal {B}}}{4}+\frac{\gamma _{\mathcal {A}\mathcal {C}}}{2}$	$\frac{1}{8}+\frac{3\gamma _{\mathcal {A}}}{16}+\frac{\gamma _{\mathcal {A}\mathcal {B}}}{4}+\frac{\gamma _{\mathcal {A}\mathcal {C}}}{2}$
3			$1+\frac{\gamma _{\mathcal {B}}}{2}$	$\gamma _{\mathcal {B}\mathcal {C}}$	$\gamma _{\mathcal {A}\mathcal {B}}$	$\frac{1}{2}+\frac{\gamma _{\mathcal {B}}}{4} + \frac{\gamma _{\mathcal {A}\mathcal {B}}}{2}$	$\frac{1}{4}+\frac{\gamma _{\mathcal {B}}}{8} + \frac{\gamma _{\mathcal {A}\mathcal {B}}}{4} +\frac{\gamma _{\mathcal {A}\mathcal {C}}}{2}$	$\frac{1}{4}+\frac{\gamma _{\mathcal {B}}}{8} + \frac{\gamma _{\mathcal {A}\mathcal {B}}}{4} +\frac{\gamma _{\mathcal {A}\mathcal {C}}}{2}$
4				$1+\frac{\gamma _{\mathcal {C}}}{2}$	$\gamma _{\mathcal {A}\mathcal {C}}$	$\frac{\gamma _{\mathcal {A}\mathcal {C}}+\gamma _{\mathcal {B}\mathcal {C}}}{2}$	$\frac{1}{2}+\frac{\gamma _{\mathcal {C}}+\gamma _{\mathcal {A}\mathcal {C}}+\gamma _{\mathcal {B}\mathcal {C}}}{4}$	$\frac{1}{2}+\frac{\gamma _{\mathcal {C}}+\gamma _{\mathcal {A}\mathcal {C}}+\gamma _{\mathcal {B}\mathcal {C}}}{4}$
5					$1+\frac{\gamma _{\mathcal {A}}}{2}$	$\frac{1}{2}+\frac{\gamma _{\mathcal {A}}}{4} + \frac{\gamma _{\mathcal {A}\mathcal {B}}}{2}$	$\frac{1}{4}+\frac{\gamma _{\mathcal {A}}}{8} + \frac{\gamma _{\mathcal {A}\mathcal {B}}}{4}+ \frac{\gamma _{\mathcal {A}\mathcal {C}}}{2}$	$\frac{1}{4}+\frac{\gamma _{\mathcal {A}}}{8} + \frac{\gamma _{\mathcal {A}\mathcal {B}}}{4}+ \frac{\gamma _{\mathcal {A}\mathcal {C}}}{2}$
6						$1+\frac{\gamma _{\mathcal {A}\mathcal {B}}}{2}$	$\frac{1}{2}+\frac{\gamma _{\mathcal {A}\mathcal {B}}+\gamma _{\mathcal {A}\mathcal {C}}+\gamma _{\mathcal {B}\mathcal {C}}}{4}$	$\frac{1}{2}+\frac{\gamma _{\mathcal {A}\mathcal {B}}+\gamma _{\mathcal {A}\mathcal {C}}+\gamma _{\mathcal {B}\mathcal {C}}}{4}$
7							$1+\frac{\gamma _{\mathcal {A}\mathcal {C}}+\gamma _{\mathcal {B}\mathcal {C}}}{4}$	$\frac{1}{2}+\frac{\gamma _{\mathcal {C}}+\gamma _{\mathcal {A}\mathcal {B}}}{8} +\frac{\gamma _{\mathcal {A}\mathcal {C}}+\gamma _{\mathcal {B}\mathcal {C}}}{4}$
8								$1+\frac{\gamma _{\mathcal {A}\mathcal {C}}+\gamma _{\mathcal {B}\mathcal {C}}}{4}$

Legarra et al. [15] proposed that pedigree relationships should be specified across all animals, and that for base animals in the pedigree, the pedigree-based relationships within and across breeds and inbreeding should be estimated from observed marker genotypes. This approach is contradictory to the García-Cortés and Toro [14] approach described above, since it violates the assumption of independence of the $\mathbf {g}^b$ and $\mathbf {g}^{b,b^{\prime }}$ vectors. The approach in which relationships are specified across breeds is named common genetic approach.

**Table 7:** Pedigree in Table 1 with metafounders

id	father	mother
$\mathcal {A}$	-	-
$\mathcal {B}$	-	-
$\mathcal {C}$	-	-
1	$\mathcal {A}$	$\mathcal {A}$
2	$\mathcal {A}$	$\mathcal {A}$
3	$\mathcal {B}$	$\mathcal {B}$
4	$\mathcal {C}$	$\mathcal {C}$
5	1	2
6	3	5
7	4	6
8	4	6

First, partial genetic and common genetic approaches for constructing pedigree-based relationships are presented, then the corresponding two different ways of constructing marker-based relationships are presented, and finally the genetic variances and covariances in model (2) are shown for the two approaches. Detailed derivations are in the “Appendix”.

### Additive genetic model for crossbred $\mathcal {C}(\mathcal {A}\mathcal {B})$ performance: partial genetic approach

For the three-way crossbreeding system, the decomposition of the additive genetic effects by García-Cortés and Toro [14] is as follows. For a $\mathcal {C}(\mathcal {A}\mathcal {B})$ crossbred animal,

$$\begin{aligned} g_i= g^{\mathcal {C}}_{\mathcal {C}(\mathcal {A}\mathcal {B}),i} + g^{\mathcal {A}}_{\mathcal {C}(\mathcal {A}\mathcal {B}),i} + g^{\mathcal {B}}_{\mathcal {C}(\mathcal {A}\mathcal {B}),i} + g^{\mathcal {A}\mathcal {B}}_{\mathcal {C}(\mathcal {A}\mathcal {B}),i}, \end{aligned}$$

where terms $\mathbf {g}^{\mathcal {C}}_{\mathcal {C}(\mathcal {A}\mathcal {B})}$, $\mathbf {g}^{\mathcal {A}}_{\mathcal {C}(\mathcal {A}\mathcal {B})}$, $\mathbf {g}^{\mathcal {B}}_{\mathcal {C}(\mathcal {A}\mathcal {B})}$ are breed of origin specific partial genetic effects and $\mathbf {g}^{\mathcal {A}\mathcal {B}}_{\mathcal {C}(\mathcal {A}\mathcal {B})}$ is a breed-segregation term. For a $\mathcal {A}\mathcal {B}$ crossbred sow,

$$\begin{aligned} g_i = g^{\mathcal {A}}_{\mathcal {A}\mathcal {B},i} + g^{\mathcal {B}}_{\mathcal {A}\mathcal {B},i}, \end{aligned}$$

with terms $\mathbf {g}^{\mathcal {A}}_{\mathcal {A}\mathcal {B}}$ and $\mathbf {g}^{\mathcal {B}}_{\mathcal {A}\mathcal {B}}$ being breed of origin partial genetic effects. Finally, for purebred animals, the three vectors of breeding values for crossbred $\mathcal {C}(\mathcal {A}\mathcal {B})$ performance, $\mathbf {g}_{\mathcal {A}}$, $\mathbf {g}_{\mathcal {B}}$ and $\mathbf {g}_{\mathcal {C}}$, are defined as being equal to the genotypic values.

In this way, a breed-specific partial genetic effect is defined for all animals containing the specific breed, and a breed-segregation partial genetic effect is defined for crossbred $\mathcal {C}(\mathcal {A}\mathcal {B})$ animals. Assuming that base individuals in the three breeds are not related across breeds implies that:

$$\begin{aligned} \mathbf {g}^{\mathcal {A}}=\left[ \begin{array}{c} \mathbf {g}_{\mathcal {A}} \\ \mathbf {g}^{\mathcal {A}}_{\mathcal {A}\mathcal {B}} \\ \mathbf {g}^{\mathcal {A}}_{\mathcal {C}(\mathcal {A}\mathcal {B})} \\ \end{array} \right] , \, \mathbf {g}^{\mathcal {B}}=\left[ \begin{array}{c} \mathbf {g}_{\mathcal {B}} \\ \mathbf {g}^{\mathcal {B}}_{\mathcal {A}\mathcal {B}} \\ \mathbf {g}^{\mathcal {B}}_{\mathcal {C}(\mathcal {A}\mathcal {B})} \\ \end{array} \right] , \, \mathbf {g}^{\mathcal {C}}=\left[ \begin{array}{c} \mathbf {g}_{\mathcal {C}} \\ \mathbf {g}^{\mathcal {C}}_{\mathcal {C}(\mathcal {A}\mathcal {B})} \\ \end{array} \right] \end{aligned}$$

are independent. In addition, for a crossbred $\mathcal {C}(\mathcal {A}\mathcal {B})$ individual the fact that it inherits either a breed $\mathcal {A}$ or $\mathcal {B}$ allele is independent of what particular alleles the $\mathcal {A}\mathcal {B}$ mother has and what alleles all other $\mathcal {A}\mathcal {B}$ individuals have, and hence $\mathbf {g}^{\mathcal {A}\mathcal {B}}_{\mathcal {C}(\mathcal {A}\mathcal {B})}$ is independent of the vectors above.

The variance-covariance matrices of the partial genetic effects become (García-Cortés and Toro [14]):

$$\begin{aligned} {\text {Var}}\left[ \begin{array}{c} \mathbf {g}_{\mathcal {A}} \\ \mathbf {g}^{\mathcal {A}}_{\mathcal {A}\mathcal {B}} \\ \mathbf {g}^{\mathcal {A}}_{\mathcal {C}(\mathcal {A}\mathcal {B})} \\ \end{array} \right] = \sigma _{g,\mathcal {A}}^2 \mathbf {A}^{\mathcal {A}}, {\text {Var}}\left[ \begin{array}{c} \mathbf {g}_{\mathcal {B}} \\ \mathbf {g}^{\mathcal {B}}_{\mathcal {A}\mathcal {B}} \\ \mathbf {g}^{\mathcal {B}}_{\mathcal {C}(\mathcal {A}\mathcal {B})} \\ \end{array} \right] = \sigma _{g,\mathcal {B}}^2 \mathbf {A}^{\mathcal {B}} , \end{aligned}$$

$$\begin{aligned} {\text {Var}}\left[ \begin{array}{c} \mathbf {g}_{\mathcal {C}} \\ \mathbf {g}^{\mathcal {C}}_{\mathcal {C}(\mathcal {A}\mathcal {B})} \\ \end{array} \right] = \sigma _{g,\mathcal {C}}^2 \mathbf {A}^{\mathcal {C}}, {\text {Var}}\left[ \mathbf {g}^{\mathcal {A}\mathcal {B}}_{\mathcal {C}(\mathcal {A}\mathcal {B})} \right] = \sigma _{g,\mathcal {A}\mathcal {B}}^2 \mathbf {A}^{\mathcal {A}\mathcal {B}}, \end{aligned}$$

where the breed-specific partial relationship matrices are defined by the recursive formulas:

$$\begin{aligned} A^{b}_{ii}&= f^{b}_i + A^{b}_{f(i)m(i)}/2, \\ A^{b}_{ii^{\prime }}&= (A^{b}_{f(i)i^{\prime }}+A^{b}_{m(i)i^{\prime }})/2, \end{aligned}$$

for breed $b=\mathcal {A},\mathcal {B},\mathcal {C}$, with $f_i^b$ denoting the breed *b* proportion, and the breed-segregation partial relationship matrix is defined by the recursive formulas:

$$\begin{aligned} A^{\mathcal {A}\mathcal {B}}_{ii}&= 2 (f^{\mathcal {A}}_{f(i)} f^{\mathcal {B}}_{f(i)} + f^{\mathcal {A}}_{m(i)} f^{\mathcal {B}}_{m(i)}) + A^{\mathcal {A}\mathcal {B}}_{f(i)m(i)}/2, \\ A^{\mathcal {A}\mathcal {B}}_{ii^{\prime }}&= (A^{\mathcal {A}\mathcal {B}}_{f(i)i^{\prime }}+A^{\mathcal {A}\mathcal {B}}_{m(i)i^{\prime }})/2, \end{aligned}$$

where in both cases non-contributing animals are not included in the resulting matrices. We immediately see that $\mathcal {C}(\mathcal {A}\mathcal {B})$ animals are the only animals contributing to matrix $\mathbf {A}^{\mathcal {A}\mathcal {B}}$, and since $f^{\mathcal {A}}_{m(i)} =f^{\mathcal {B}}_{m(i)}=1/2$ for these animals, the matrix is a diagonal matrix with diagonal elements equal to $2\times 1/2\times 1/2 = 1/2$. This specification of additive relationships using partial relationship matrices is equivalent to the specification in Eqs. (3) and (4).

To illustrate the different partial relationship matrices, we analysed the small pedigree in Table 1. Tables 2, 3, 4 and 5 show the partial relationship matrices for this example.

Wei and van der Werf [8] presented a reduced form of the two-way crossbreeding model (1) in which the Mendelian sampling term of the genetic effect on crossbred animals was included in the residual error term. A reduced model can also be formulated for the three-way crossbreeding model by expressing:

$$\begin{aligned} g_i&= 0.5g_{f(i)} + 0.5g_{m(i)} + \Phi _{\mathcal {C}(\mathcal {A}\mathcal {B}),i} \\&= 0.5g_{\mathcal {C},f(i)} + 0.5 g^{\mathcal {A}}_{\mathcal {A}\mathcal {B},m(i)} + 0.5g^{\mathcal {B}}_{\mathcal {A}\mathcal {B},m(i)} + \Phi _{\mathcal {C}(\mathcal {A}\mathcal {B}),i}, \end{aligned}$$

for a $\mathcal {C}(\mathcal {A}\mathcal {B})$ crossbred animal *i*, where $\Phi _{\mathcal {C}(\mathcal {A}\mathcal {B}),i}$ is the Mendelian sampling term. The Mendelian sampling terms are independent among the $\mathcal {C}(\mathcal {A}\mathcal {B})$ crossbred animals, and by making the approximation that father *f*(*i*) is not inbred and since mother *m*(*i*) is not inbred, the variance is constant. In this way, the Mendelian sampling error term can be included into the residual error term $\mathbf {e}_{\mathcal {C}(\mathcal {A}\mathcal {B})}$ in model (2), and the model can be formulated using three breed-specific partial relationship matrices defined on the $\mathcal {A},\mathcal {B},\mathcal {C}$ and $\mathcal {A}\mathcal {B}$ animals. However, as explained in Christensen et al. [11], such a reduced model cannot be extended to incorporate marker genotypes since these provide information about the Mendelian sampling term. Therefore, we did not pursue the reduced form of the model any further.

Note that model (2) with relationships as presented here is the most obvious generalisation of the Wei and van der Werf model in Eq. (1) from two to three breeds since base individuals are assumed unrelated. Without a formulation using partial relationship matrices, it would be difficult to estimate parameters in this model using standard animal breeding software.

### Additive genetic model for crossbred $\mathcal {C}(\mathcal {A}\mathcal {B})$ performance: common genetic approach

In the previous subsection, base animals were assumed to be unrelated. An alternative proposed by Legarra et al. [15] is to assume that base animals are related and inbred within breeds and related between breeds with relationships determined by:

$$\begin{aligned} \varvec{\Gamma }= \left[ \begin{array}{ccc} \gamma _{\mathcal {A}} &{} \gamma _{\mathcal {A},\mathcal {B}} &{} \gamma _{\mathcal {A},\mathcal {C}} \\ \gamma _{\mathcal {A},\mathcal {B}} &{} \gamma _{\mathcal {B}} &{} \gamma _{\mathcal {B},\mathcal {C}} \\ \gamma _{\mathcal {A},\mathcal {C}} &{} \gamma _{\mathcal {B},\mathcal {C}} &{} \gamma _{\mathcal {C}} \\ \end{array} \right] . \end{aligned}$$

This means that among the base animals, the variance-covariance of genetic effects is as follows. The variance-covariance within breed is defined by:

$$\begin{aligned} {\text {Var}}(g_i)= \sigma _g^2(1 + \gamma _b/2), \end{aligned}$$

for an individual in breed *b*, and

$$\begin{aligned} {\text {Cov}}(g_i,g_{i^{\prime }})= \sigma _g^2\gamma _b, \end{aligned}$$

for two individuals in breed *b*, i.e. base animals are inbred with coefficient $\gamma _b/2$ and related with relationship coefficient $\gamma _b$. Furthermore,

$$\begin{aligned} {\text {Cov}}(g_i,g_{i^{\prime }})= \sigma _g^2 \gamma _{b,b^{\prime }}, \end{aligned}$$

for two individuals in different breeds *b* and $b^{\prime }$, i.e. base animals in different breeds are related. Therefore, a joint relationship matrix is specified among all base animals, and by applying the usual recursive definition:

$$\begin{aligned} A(\varvec{\Gamma })_{ii}&= 1 + A(\varvec{\Gamma })_{f(i)m(i)}/2, \\ A(\varvec{\Gamma })_{ii^{\prime }}&= (A(\varvec{\Gamma })_{f(i)i^{\prime }}+A(\varvec{\Gamma })_{m(i)i^{\prime }})/2, \end{aligned}$$

an additive relationship matrix $\mathbf {A}(\varvec{\Gamma })$ is defined across all animals with relationships among the three base populations $\mathcal {A},\mathcal {B}$ and $\mathcal {C}$ defined by matrix $\varvec{\Gamma }$. The variance-covariance of genetic effects is therefore determined by

5 $$\begin{aligned} {\text {Var}}\left[ \begin{array}{c} \mathbf {g}_{\mathcal {A}} \\ \mathbf {g}_{\mathcal {B}} \\ \mathbf {g}_{\mathcal {C}} \\ \mathbf {g}_{\mathcal {A}\mathcal {B}} \\ \mathbf {g}_{\mathcal {C}(\mathcal {A}\mathcal {B})} \\ \end{array} \right] = \sigma ^2_g \mathbf {A}(\varvec{\Gamma }). \end{aligned}$$

Table 6 shows the common relationship matrix for the pedigree in Table 1.

Legarra et al. [15] suggested a framework where individuals in the base population of the pedigree are related because they originate from overlapping ancestral populations with a finite size, and they termed each of these ancestral populations as a meta-founder to be included in the pedigree. Here, $\mathcal {A},\mathcal {B},\mathcal {C}$ are meta-founders, and each base individual in the pedigree has a meta-founder, which is both its parents; see example in Table 7. When extending the pedigree and the matrix $\mathbf {A}(\varvec{\Gamma })$ with these meta-founders, Legarra et al. [15] showed that the algorithms for computing the sparse inverse matrix $\mathbf {A}(\varvec{\Gamma })^{-1}$ directly as in Henderson [16] and submatrices of $\mathbf {A}(\varvec{\Gamma })$ by the Colleau algorithm [17] are as usual.

The parameter $\sigma _g^2$ in Eq. (5) does not correspond to the usual genetic variance which is the variance among unrelated individuals in the base population. As explained in Legarra et al. [15], $\sigma _g^2(1 - \gamma _b/2)$ corresponds to the variance among unrelated breed *b* animals, and therefore the genetic variances for crossbred $\mathcal {C}(\mathcal {A}\mathcal {B})$ performance are $\sigma _g^2(1 - \gamma _{\mathcal {A}}/2)$, $\sigma _g^2(1 - \gamma _{\mathcal {B}}/2)$ and $\sigma _g^2(1 - \gamma _{\mathcal {C}}/2)$, corresponding to $\sigma _{g,\mathcal {A}}^2$, $\sigma _{g,\mathcal {B}}^2$ and $\sigma _{g,\mathcal {C}}^2$ in the previous section, respectively. In addition, Legarra et al. [15] explained that the breed-segregation variance is $\sigma ^2_g((\gamma _{\mathcal {A}}+\gamma _{\mathcal {B}})/2-\gamma _{\mathcal {A},\mathcal {B}})/4$, which corresponds to $\sigma _{g,\mathcal {A}\mathcal {B}}^2$ in the previous section.

### Genomic model for crossbred $\mathcal {C}(\mathcal {A}\mathcal {B})$ performance: partial genetic approach

Marker-based partial relationship matrices are constructed by tracing breed of origin of alleles and defining relationships according to breed of origin. Assume that breed of origin of alleles can be determined for all animals and define breed-specific allele content matrices as: matrix $\mathbf {m}^b$ with entries 0, 1, 2 for purebred *b* animals, matrices $\mathbf {z}^{\mathcal {A}}$ and $\mathbf {z}^{\mathcal {B}}$ with entries 0, 1 for paternal and maternal alleles, respectively, for crossbred $\mathcal {A}\mathcal {B}$ animals, matrix $\mathbf {z}^{\mathcal {C}}$ with entries 0, 1 for paternal allele of crossbred $\mathcal {C}(\mathcal {A}\mathcal {B})$ animals, and finally matrices $\mathbf {z}_p^{\mathcal {A}}$ and $\mathbf {z}_p^{\mathcal {B}}$ with entries 0, 1, respectively, for crossbred $\mathcal {C}(\mathcal {A}\mathcal {B})$ animals when the breed-specific allele is inherited and zero otherwise. This means that breed of origin of each allele needs to be traced, usually by a phasing software [18].

Marker-based breed-specific partial relationship matrices are constructed as follows (details can be found in the “Appendix”). For breed $\mathcal {A}$, the marker-based breed $\mathcal {A}$ specific partial relationship matrix $\mathbf {G}^{\mathcal {A}}$ is divided into submatrices with indices denoting genotyped breed $\mathcal {A}$ and crossbred $\mathcal {A}\mathcal {B}$ animals,

$$\begin{aligned} \mathbf {G}^{\mathcal {A}} = \left[ \begin{array}{ccc} \mathbf {G}^{\mathcal {A}}_{\mathcal {A},\mathcal {A}} &{} \mathbf {G}^{\mathcal {A}}_{\mathcal {A},\mathcal {A}\mathcal {B}} &{} \mathbf {G}^{\mathcal {A}}_{\mathcal {A},\mathcal {C}(\mathcal {A}\mathcal {B})} \\ \mathbf {G}^{\mathcal {A}}_{\mathcal {A}\mathcal {B},\mathcal {A}} &{} \mathbf {G}^{\mathcal {A}}_{\mathcal {A}\mathcal {B},\mathcal {A}\mathcal {B}} &{} \mathbf {G}^{\mathcal {A}}_{\mathcal {A}\mathcal {B},\mathcal {C}(\mathcal {A}\mathcal {B})} \\ \mathbf {G}^{\mathcal {A}}_{\mathcal {C}(\mathcal {A}\mathcal {B}),\mathcal {A}} &{} \mathbf {G}^{\mathcal {A}}_{\mathcal {C}(\mathcal {A}\mathcal {B}),\mathcal {A}\mathcal {B}} &{} \mathbf {G}^{\mathcal {A}}_{\mathcal {C}(\mathcal {A}\mathcal {B}),\mathcal {C}(\mathcal {A}\mathcal {B})} \end{array} \right] , \end{aligned}$$

which are defined as

$$\begin{aligned}&\mathbf {G}^{\mathcal {A}}_{\mathcal {A},\mathcal {A}}=\frac{(\mathbf {m}^{\mathcal {A}}-2\mathbf {p}^{\mathcal {A}}\mathbf {1}^\mathrm{{\scriptstyle {T}}})(\mathbf {m}^{\mathcal {A}}-2\mathbf {p}^{\mathcal {A}}\mathbf {1}^\mathrm{{\scriptstyle {T}}})^\mathrm{{\scriptstyle {T}}}}{s^{\mathcal {A}}} , \\&\mathbf {G}^{\mathcal {A}}_{\mathcal {A},\mathcal {A}\mathcal {B}}=\frac{(\mathbf {m}^{\mathcal {A}}-2\mathbf {p}^{\mathcal {A}}\mathbf {1}^\mathrm{{\scriptstyle {T}}})(\mathbf {z}^{\mathcal {A}}-\mathbf {p}^{\mathcal {A}}\mathbf {1}^\mathrm{{\scriptstyle {T}}})^\mathrm{{\scriptstyle {T}}}}{s^{\mathcal {A}}} , \\&\mathbf {G}^{\mathcal {A}}_{\mathcal {A},\mathcal {C}(\mathcal {A}\mathcal {B})}=\frac{(\mathbf {m}^{\mathcal {A}}-2\mathbf {p}^{\mathcal {A}}\mathbf {1}^\mathrm{{\scriptstyle {T}}})(\mathbf {z}_p^{\mathcal {A}}-\mathbf {p}_p^{\mathcal {A}})^\mathrm{{\scriptstyle {T}}}}{s^{\mathcal {A}}} , \\&\mathbf {G}^{\mathcal {A}}_{\mathcal {A}\mathcal {B},\mathcal {A}\mathcal {B}}=\frac{(\mathbf {z}^{\mathcal {A}}-\mathbf {p}^{\mathcal {A}}\mathbf {1}^\mathrm{{\scriptstyle {T}}})(\mathbf {z}^{\mathcal {A}}-\mathbf {p}^{\mathcal {A}}\mathbf {1}^\mathrm{{\scriptstyle {T}}})^\mathrm{{\scriptstyle {T}}}}{s^{\mathcal {A}}}, \\&\mathbf {G}^{\mathcal {A}}_{\mathcal {A}\mathcal {B},\mathcal {C}(\mathcal {A}\mathcal {B})}=\frac{(\mathbf {z}^{\mathcal {A}}-\mathbf {p}^{\mathcal {A}}\mathbf {1}^\mathrm{{\scriptstyle {T}}})(\mathbf {z}_p^{\mathcal {A}}-\mathbf {p}_p^{\mathcal {A}})^\mathrm{{\scriptstyle {T}}}}{s^{\mathcal {A}}} , \\&\mathbf {G}^{\mathcal {A}}_{\mathcal {C}(\mathcal {A}\mathcal {B}),\mathcal {C}(\mathcal {A}\mathcal {B})}=\frac{(\mathbf {z}_p^{\mathcal {A}}-\mathbf {p}_p^{\mathcal {A}})(\mathbf {z}_p^{\mathcal {A}}-\mathbf {p}_p^{\mathcal {A}})^\mathrm{{\scriptstyle {T}}}}{s^{\mathcal {A}}} , \end{aligned}$$

where the vector $\mathbf {p}^{\mathcal {A}}$ contains breed $\mathcal {A}$ specific allele frequencies, matrix $\mathbf {p}_p^{\mathcal {A}}$ has elements (*i*, *j*) equal to $p^{\mathcal {A}}_j$ when the crossbred $\mathcal {C}(\mathcal {A}\mathcal {B})$ individual *i* inherited an $\mathcal {A}$ specific allele and zero otherwise, and $s^{\mathcal {A}}$ is a scaling parameter. The marker-based breed $\mathcal {B}$ specific partial relationship matrix $\mathbf {G}^{\mathcal {B}}$ is defined similarly to $\mathbf {G}^{\mathcal {A}}$, and the marker-based breed $\mathcal {C}$ specific partial relationship matrix is

$$\begin{aligned} \mathbf {G}^{\mathcal {C}} = \left[ \begin{array}{cc} \mathbf {G}^{\mathcal {C}}_{\mathcal {C},\mathcal {C}} &{} \mathbf {G}^{\mathcal {A}}_{\mathcal {C},\mathcal {C}(\mathcal {A}\mathcal {B})} \\ \mathbf {G}^{\mathcal {C}}_{\mathcal {C}(\mathcal {A}\mathcal {B}),\mathcal {C}} &{} \mathbf {G}^{\mathcal {A}}_{\mathcal {C}(\mathcal {A}\mathcal {B}),\mathcal {C}(\mathcal {A}\mathcal {B})} \end{array} \right] , \end{aligned}$$

where submatrices are defined as

$$\begin{aligned}&\mathbf {G}^{\mathcal {C}}_{\mathcal {C},\mathcal {C}}=\frac{(\mathbf {m}^{\mathcal {C}}-2\mathbf {p}^{\mathcal {C}}\mathbf {1}^\mathrm{{\scriptstyle {T}}})(\mathbf {m}^{\mathcal {C}}-2\mathbf {p}^{\mathcal {C}}\mathbf {1}^\mathrm{{\scriptstyle {T}}})^\mathrm{{\scriptstyle {T}}}}{s^{\mathcal {C}}} , \\&\mathbf {G}^{\mathcal {C}}_{\mathcal {C},\mathcal {C}(\mathcal {A}\mathcal {B})}=\frac{(\mathbf {m}^{\mathcal {C}}-2\mathbf {p}^{\mathcal {C}}\mathbf {1}^\mathrm{{\scriptstyle {T}}})(\mathbf {z}^{\mathcal {C}}-\mathbf {p}^{\mathcal {A}}\mathbf {1}^\mathrm{{\scriptstyle {T}}})^\mathrm{{\scriptstyle {T}}}}{s^{\mathcal {C}}} , \\&\mathbf {G}^{\mathcal {C}}_{\mathcal {C}(\mathcal {A}\mathcal {B}),\mathcal {C}(\mathcal {A}\mathcal {B})}=\frac{(\mathbf {z}^{\mathcal {C}}-\mathbf {p}^{\mathcal {C}}\mathbf {1}^\mathrm{{\scriptstyle {T}}})(\mathbf {z}^{\mathcal {C}}-\mathbf {p}^{\mathcal {C}}\mathbf {1}^\mathrm{{\scriptstyle {T}}})^\mathrm{{\scriptstyle {T}}}}{s^{\mathcal {C}}} , \end{aligned}$$

where the vector $\mathbf {p}^{\mathcal {C}}$ contains estimated breed $\mathcal {C}$ specific allele frequencies and $s^{\mathcal {C}}$ is a scaling parameter.

The breed-segregation partial relationship matrix is defined as:

$$\begin{aligned} G^{\mathcal {A}\mathcal {B}}_{i,i^{\prime }} = \sum _j r_{d^j_i} r_{d^j_{i^{\prime }}}/(2n), \end{aligned}$$

where $r_{d^j_i}=1$ when $d^j_i\in \mathcal {A}$ and $r_{d^j_i}=-1$ when $d^j_i\in \mathcal {B}$, $r_{d^j_{i^{\prime }}}$ is defined similarly to $r_{d^j_i}$, and *n* is the number of markers. Note that diagonal elements of $\mathbf {G}^{\mathcal {A}\mathcal {B}}$ equal diagonal elements of $\mathbf {A}^{\mathcal {A}\mathcal {B}}$ (i.e. 1/2). Off-diagonal elements of $G^{\mathcal {A}\mathcal {B}}$ measure whether pairs of individuals share more alleles from a particular parental breed ($\mathcal {A}$ or $\mathcal {B}$) than expected. Expectations of off-diagonal elements $\mathbf {G}^{\mathcal {A}\mathcal {B}}$ equal off-diagonal elements of $\mathbf {A}^{\mathcal {A}\mathcal {B}}$ (i.e. 0).

Relationship matrices that combine pedigree and marker information [2, 4] can then be constructed. Below, indices 1 and 2 in submatrices denote non-genotyped and genotyped animals, respectively. The breed $b=\mathcal {A},\mathcal {B},\mathcal {C}$ specific combined relationship matrices are given by their sparse inverses

6 $$\begin{aligned} (\mathbf {H}^{b})^{-1} = \left[ \begin{array}{cc} \mathbf {0}&{} \mathbf {0}\\ \mathbf {0}&{} (\mathbf {G}^{b})^{-1} - (\mathbf {A}^{b}_{22})^{-1} \end{array} \right] + (\mathbf {A}^{b})^{-1}, \end{aligned}$$

for $b=\mathcal {A},\mathcal {B},\mathcal {C}$, and because $\mathbf {A}^{\mathcal {A}\mathcal {B}}=\mathbf {I}/2$ the breed-segregation combined relationship matrix is

7 $$\begin{aligned} \mathbf {H}^{\mathcal {A}\mathcal {B}} = \left[ \begin{array}{cc} \mathbf {I}/2 &{} \mathbf {0}\\ \mathbf {0}&{} \mathbf {G}^{\mathcal {A}\mathcal {B}} \end{array} \right] . \end{aligned}$$

Matrices $(\mathbf {A}^{\mathcal {A}})^{-1}$, $(\mathbf {A}^{\mathcal {B}})^{-1}$ and $(\mathbf {A}^{\mathcal {C}})^{-1}$ can be computed directly in sparse format and matrices $\mathbf {A}^{\mathcal {A}}_{22}$, $\mathbf {A}^{\mathcal {B}}_{22}$ and $\mathbf {A}^{\mathcal {C}}_{22}$ can be computed by the Colleau algorithm [17]; see Christensen et al. [11].

The breed-specific partial marker-based relationship matrices above require estimates of breed-specific allele frequencies. Such estimates can be obtained from marker genotypes of purebred animals and breed-specific marker alleles for crossbred animals. Furthermore, there is a need to adjust these matrices to be compatible with partial pedigree relationship matrices similar to Christensen et al. [11, 19], i.e. $\mathbf {G}^b_a=\mathbf {G}^b\beta _b+\alpha _b\mathbf {J}^b$ where $\alpha _b$ and $\beta _b$ are parameters and $\mathbf {J}^b$ is a matrix with entries $\mathbf {J}^b_{i,i^{\prime }}=f^b_i f^b_{i^{\prime }}$. The scaling parameters $s^b$ in marker-based relationship matrices $\mathbf {G}^{b}$, b=$\mathcal {A},\mathcal {B},\mathcal {C}$ are unspecified above, since the compatibility adjustment involves a scaling parameter $\beta _b$ for each breed, and therefore $s^b$ can be arbitrary. On the other hand, matrix $\mathbf {G}^{\mathcal {A}\mathcal {B}}$ does not need an adjustment.

Finally, to incorporate the fact that marker genotypes only capture a fraction of the genetic effects, the partial marker-based relationship matrices $\mathbf {G}^{b}$, $b\in \mathcal {A},\mathcal {B},\mathcal {C}$ and $\mathbf {G}^{\mathcal {A}\mathcal {B}}$ above may be replaced by matrices $\mathbf {G}^{b}_{\omega }=\mathbf {G}^{b}(1-\omega )+\mathbf {A}^{b}\omega$, $b\in \mathcal {A},\mathcal {B},\mathcal {C}$ and $\mathbf {G}^{\mathcal {A}\mathcal {B}}(1-\omega )+\mathbf {A}^{\mathcal {A}\mathcal {B}}\omega$, respectively, where $\omega$ is the fraction of genetic variance not captured by marker genotypes [4].

### Genomic model for crossbred $\mathcal {C}(\mathcal {A}\mathcal {B})$ performance: common genetic approach

The marker-based relationship matrix is constructed as usual across all genotyped animals:

$$\begin{aligned} \mathbf {G}=\frac{(\mathbf {m}-\mathbf {1}\mathbf {1}^\mathrm{{\scriptstyle {T}}})(\mathbf {m}-\mathbf {1}\mathbf {1}^\mathrm{{\scriptstyle {T}}})^\mathrm{{\scriptstyle {T}}}}{s} , \end{aligned}$$

where $\mathbf {m}$ is the gene content matrix with entries 0, 1, 2 and *s* is scaling parameter. As in Christensen [20] and Legarra et al. [15], we chose common allele frequencies, i.e. $p_j=0.5$, and then determine the parameters in matrix $\varvec{\Gamma }$ and parameter *s* such that the pedigree-based and marker-based relationship matrices are compatible. Parameters in matrix $\varvec{\Gamma }$ and scaling parameter *s* can be estimated by matching $\mathbf {A}(\varvec{\Gamma })$ and $\mathbf {G}$ for purebred individuals; see Legarra et al. [15]. For example, if genotyping is done in each of the three pure breeds then the following system of equations can be used to determine the parameters:

$$\begin{aligned} \bar{G}_{\mathcal {A},\mathcal {B}}/s&=\gamma _{\mathcal {A},\mathcal {B}}, \ \ \bar{G}_{\mathcal {A},\mathcal {C}}/s=\gamma _{\mathcal {A},\mathcal {C}}, \ \ \bar{G}_{\mathcal {B},\mathcal {C}}/s=\gamma _{\mathcal {B},\mathcal {C}}, \\ \bar{G}_{\mathcal {A},\mathcal {A}}/s&=\bar{A}_{\mathcal {A},\mathcal {A}}(1-\gamma _{\mathcal {A}}/2) + \gamma _{\mathcal {A}} , \\ \bar{G}_{\mathcal {B},\mathcal {B}}/s&= \bar{A}_{\mathcal {B},\mathcal {B}}(1-\gamma _{\mathcal {B}}/2) + \gamma _{\mathcal {B}}, \\ \bar{G}/s&=(\bar{\text {diag}(A_{\mathcal {A},\mathcal {A}})}(1-\gamma _{\mathcal {A}}/2) + \gamma _{\mathcal {A}})/3 \\&\quad +(\bar{\text {diag}(A_{\mathcal {B},\mathcal {B}})}(1-\gamma _{\mathcal {B}}/2) + \gamma _{\mathcal {B}})/3 \\&\quad +(\bar{\text {diag}(A_{\mathcal {C},\mathcal {C}})}(1-\gamma _{\mathcal {C}}/2) + \gamma _{\mathcal {C}})/3, \end{aligned}$$

where $\bar{G}_{\mathcal {A},\mathcal {A}}$, $\bar{G}_{\mathcal {A},\mathcal {B}}$, $\bar{G}_{\mathcal {A},\mathcal {C}}$, $\bar{G}_{\mathcal {B},\mathcal {B}}$, $\bar{G}_{\mathcal {B},\mathcal {C}}$ and $\bar{G}_{\mathcal {C},\mathcal {C}}$ denote averages of elements in submatrices of $\mathbf {G}$, $\bar{G}=(\bar{G}_{\mathcal {A},\mathcal {A}}+\bar{G}_{\mathcal {B},\mathcal {B}}+\bar{G}_{\mathcal {C},\mathcal {C}})/3$, $\bar{A}_{\mathcal {A},\mathcal {A}}$, $\bar{A}_{\mathcal {A},\mathcal {B}}$, $\bar{A}_{\mathcal {A},\mathcal {C}}$, $\bar{A}_{\mathcal {B},\mathcal {B}}$, $\bar{A}_{\mathcal {B},\mathcal {C}}$ and $\bar{A}_{\mathcal {C},\mathcal {C}}$ denote averages of elements in submatrices of $\mathbf {A}_{22}$, and $\bar{\text {diag}(A_{\mathcal {A},\mathcal {A}})}$, $\bar{\text {diag}(A_{\mathcal {B},\mathcal {B}})}$, $\bar{\text {diag}(A_{\mathcal {C},\mathcal {C}})}$ denote averages of diagonal elements in submatrices of $\mathbf {A}_{22}$. This is a linear system of 7 equations with 7 parameters $\gamma _{\mathcal {A}}, \gamma _{\mathcal {B}}, \gamma _{\mathcal {C}}, \gamma _{\mathcal {A},\mathcal {B}}, \gamma _{\mathcal {A},\mathcal {C}}, \gamma _{\mathcal {B},\mathcal {C}}$ and 1/*s* and can therefore be solved directly to obtain estimates.

The relationship matrix that combines pedigree and marker information becomes

8 $$\begin{aligned} \mathbf {H}(\varvec{\Gamma })^{-1} = \left[ \begin{array}{cc} \mathbf {0}&{} \mathbf {0}\\ \mathbf {0}&{} \mathbf {G}^{-1} - (\mathbf {A}(\varvec{\Gamma })_{22})^{-1} \end{array} \right] + \mathbf {A}(\varvec{\Gamma })^{-1} . \end{aligned}$$

Finally, similar to the previous section, the marker-based relationship matrices $\mathbf {G}$ above may be replaced by $\mathbf {G}_{\omega }=\mathbf {G}(1-\omega )+\mathbf {A}(\varvec{\Gamma })\omega$ where $\omega$ is the fraction of genetic variance that is not captured by marker genotypes.

### Genetic models for both purebred and crossbred $\mathcal {C}(\mathcal {A}\mathcal {B})$ performances

In the previous sections, partial genetic and common genetic models for additive genetic effects for crossbred $\mathcal {C}(\mathcal {A}\mathcal {B})$ performance were presented, and in both cases genomic versions of the models and combined relationship matrices were shown. Now, we show how the genetic variances and covariances for the model in Eq. (2) look like in the two cases.

For the partial genetic case, the vector of genetic effects on crossbred $\mathcal {C}(\mathcal {A}\mathcal {B})$ individuals equals $\mathbf {g}^{\mathcal {C}}_{\mathcal {C}(\mathcal {A}\mathcal {B})} + \mathbf {g}^{\mathcal {A}}_{\mathcal {C}(\mathcal {A}\mathcal {B})} + \mathbf {g}^{\mathcal {B}}_{\mathcal {C}(\mathcal {A}\mathcal {B})} + \mathbf {g}^{\mathcal {A}\mathcal {B}}_{\mathcal {C}(\mathcal {A}\mathcal {B})}$ and based on $\mathbf {g}^{\mathcal {A}}_{\mathcal {C}(\mathcal {A}\mathcal {B})}$, $\mathbf {g}^{\mathcal {B}}_{\mathcal {C}(\mathcal {A}\mathcal {B})}$ and $\mathbf {g}^{\mathcal {C}}_{\mathcal {C}(\mathcal {A}\mathcal {B})}$, breed-specific partial relationships define the breeding values for crossbred $\mathcal {C}(\mathcal {A}\mathcal {B})$ performance on purebred animals, $\mathbf {g}_{\mathcal {A}}$, $\mathbf {g}_{\mathcal {B}}$ and $\mathbf {g}_{\mathcal {C}}$, respectively. Combining these effects with the breeding values for purebred performances, $\mathbf {a}_{\mathcal {A}}$, $\mathbf {a}_{\mathcal {B}}$ and $\mathbf {a}_{\mathcal {C}}$, the variance-covariance of genetic effects is determined by

$$\begin{aligned}&{\text {Var}}\left[ \begin{array}{c} \mathbf {a}_{\mathcal {A}} \\ \star \\ \star \\ \text{- - - - -} \\ \mathbf {g}_{\mathcal {A}} \\ \mathbf {g}^{\mathcal {A}}_{\mathcal {A}\mathcal {B}} \\ \mathbf {g}^{\mathcal {A}}_{\mathcal {C}(\mathcal {A}\mathcal {B})} \\ \end{array} \right] = \varvec{\Sigma }_{\mathcal {A}}\bigotimes \mathbf {H}^{\mathcal {A}}, \\&{\text {Var}}\left[ \begin{array}{c} \mathbf {a}_{\mathcal {B}} \\ \star \\ \star \\ \text{- - - - -} \\ \mathbf {g}_{\mathcal {B}} \\ \mathbf {g}^{\mathcal {B}}_{\mathcal {A}\mathcal {B}} \\ \mathbf {g}^{\mathcal {B}}_{\mathcal {C}(\mathcal {A}\mathcal {B})} \\ \end{array} \right] = \varvec{\Sigma }_{\mathcal {B}} \bigotimes \mathbf {H}^{\mathcal {B}} , \\&{\text {Var}}\left[ \begin{array}{c} \mathbf {a}_{\mathcal {C}} \\ \star \\ \text{- - - - -} \\ \mathbf {g}_{\mathcal {C}} \\ \mathbf {g}^{\mathcal {C}}_{\mathcal {C}(\mathcal {A}\mathcal {B})} \\ \end{array} \right] = \varvec{\Sigma }_{\mathcal {C}} \bigotimes \mathbf {H}^{\mathcal {C}}, \\&{\text {Var}}\left[ \mathbf {g}^{\mathcal {A}\mathcal {B}}_{\mathcal {C}(\mathcal {A}\mathcal {B})} \right] = \sigma _{g,\mathcal {A}\mathcal {B}}^2 \mathbf {H}^{\mathcal {A}\mathcal {B}}, \end{aligned}$$

with the four vectors being independent. Here, $\bigotimes$ denotes the Kronecker product, $\star$ denotes artificial random vectors such that the genetic variance-covariance matrices can be expressed using Kronecker products and matrices

$$\begin{aligned} \varvec{\Sigma }_b = \left[ \begin{array}{cc} \sigma ^2_{a,b} &{} \sigma _{ag,b} \\ \sigma _{ag,b} &{} \sigma ^2_{g,b} \end{array} \right] , \end{aligned}$$

for $b=\mathcal {A},\mathcal {B}, \mathcal {C}$, are the $2\times 2$ variance-covariance matrices containing the genetic variances for purebred breeding values and crossbred breeding values, and the covariance between them. Thus, using partial relationship matrices provides a formulation of the model in Eq. (2) using Kronecker products, such that parameters can be estimated and breeding values predicted using standard animal breeding software. In this model, there are 10 genetic parameters and $2(n_{\mathcal {A}}+ n_{\mathcal {B}} + n_{\mathcal {C}} + n_{\mathcal {A}\mathcal {B}}) + 3 n_{\mathcal {C}(\mathcal {A}\mathcal {B})}$ genetic values where $n_{\mathcal {X}}$ is the number of individuals in population $\mathcal {X}$.

For the common genetic case, all individuals are related, and breeding values for crossbred $\mathcal {C}(\mathcal {A}\mathcal {B})$ performance on purebred animals, $\mathbf {g}_{\mathcal {A}}$, $\mathbf {g}_{\mathcal {B}}$ and $\mathbf {g}_{\mathcal {C}}$, are defined by additive relationships to the genetic effects on crossbreds, $\mathbf {g}_{\mathcal {C}(\mathcal {A}\mathcal {B})}$. Combining these effects with the breeding values for purebred performances, $\mathbf {a}_{\mathcal {A}}$, $\mathbf {a}_{\mathcal {B}}$ and $\mathbf {a}_{\mathcal {C}}$, the variance-covariance of genetic effects equals:

$$\begin{aligned} {\text {Var}}\left[ \begin{array}{c} \mathbf {a}_{\mathcal {A}} \\ \star \\ \star \\ \star \\ \star \\ \text{- - - - -} \\ \star \\ \mathbf {a}_{\mathcal {B}} \\ \star \\ \star \\ \star \\ \text{- - - - -} \\ \star \\ \star \\ \mathbf {a}_{\mathcal {C}} \\ \star \\ \star \\ \text{- - - - -} \\ \mathbf {g}_{\mathcal {A}} \\ \mathbf {g}_{\mathcal {B}} \\ \mathbf {g}_{\mathcal {C}} \\ \mathbf {g}_{\mathcal {A}\mathcal {B}} \\ \mathbf {g}_{\mathcal {C}(\mathcal {A}\mathcal {B})} \\ \end{array} \right] = \varvec{\Sigma }\bigotimes \mathbf {H}(\varvec{\Gamma }), \end{aligned}$$

where $\star$ denotes artificial random vectors and $\varvec{\Sigma }$ is the $4\times 4$ genetic variance-covariance matrix:

$$\begin{aligned} \varvec{\Sigma }= \left[ \begin{array}{cccc} \sigma ^2_{a,\mathcal {A}} &{} \sigma _{a,\mathcal {A},\mathcal {B}} &{} \sigma _{a,\mathcal {A},\mathcal {C}} &{} \sigma _{ag,\mathcal {A}} \\ \sigma _{a,\mathcal {A},\mathcal {B}} &{} \sigma ^2_{a,\mathcal {B}} &{} \sigma _{a,\mathcal {B},\mathcal {C}} &{} \sigma _{ag,\mathcal {B}} \\ \sigma _{a,\mathcal {A},\mathcal {C}} &{} \sigma _{a,\mathcal {B},\mathcal {C}} &{} \sigma ^2_{a,\mathcal {C}} &{} \sigma _{ag,\mathcal {C}} \\ \sigma _{ag,\mathcal {A}} &{} \sigma _{ag,\mathcal {B}} &{} \sigma _{ag,\mathcal {C}} &{} \sigma ^2_g \\ \end{array} \right] . \end{aligned}$$

The formulation of the model in Eq. (2) using Kronecker products implies that parameters can be estimated and breeding values predicted using standard animal breeding software. This model contains 10 genetic parameters and $2(n_{\mathcal {A}}+ n_{\mathcal {B}} + n_{\mathcal {C}}) + n_{\mathcal {A}\mathcal {B}}) + n_{\mathcal {C}(\mathcal {A}\mathcal {B})}$ genetic values, and in addition, 6 parameters in matrix $\varvec{\Gamma }$.

In the common genetic case, there are three parameters $\sigma _{a,\mathcal {A},\mathcal {B}}$, $\sigma _{a,\mathcal {A},\mathcal {C}}$ and $\sigma _{a,\mathcal {B},\mathcal {C}}$ which are genetic covariances between purebred performances, and these parameters are not present in the partial genetic case. The reason is that they would not be identifiable since there is no specification of the relationships across breeds in the partial genetic case. In the common genetic case, the identifiability of $\sigma _{a,\mathcal {A},\mathcal {B}}$, $\sigma _{a,\mathcal {A},\mathcal {C}}$ and $\sigma _{a,\mathcal {B},\mathcal {C}}$ relies on the genomic relationships between pairs of animals in different breeds. In the partial genetic case, there are four genetic parameters for crossbred performance, $\sigma _{g,\mathcal {A}}^2$, $\sigma _{g,\mathcal {B}}^2$, $\sigma _{g,\mathcal {C}}^2$ and $\sigma _{g,\mathcal {A}\mathcal {B}}^2$ that scale each of the four partial relationship matrices, whereas in the common genetic case there is only one such parameter $\sigma _g^2$. As explained in a previous section, there is a correspondence between these parameters via the parameters in matrix $\varvec{\Gamma }$ as follows: $\sigma _{g,b}^2=\sigma _g^2(1 - \gamma _b/2)$, $b=\mathcal {A},\mathcal {B},\mathcal {C}$, $\sigma ^2_{g,\mathcal {A}\mathcal {B}}=\sigma ^2_g((\gamma _{\mathcal {A}}+\gamma _{\mathcal {B}})/2-\gamma _{\mathcal {A},\mathcal {B}})/4$. However, note that there is a difference between estimating $\sigma _{g,\mathcal {A}}^2$, $\sigma _{g,\mathcal {B}}^2$, $\sigma _{g,\mathcal {C}}^2$ and $\sigma _{g,\mathcal {A}\mathcal {B}}^2$ from phenotypes as in the partial genetic case, and determining these from a general $\sigma ^2_g$ and parameters in $\varvec{\Gamma }$, which are estimated based on marker genotypes as in the common genetic case.

## Discussion

For three-way crossbreeding, we presented models based on both pedigree-based, marker-based and combined relationships. Using combined relationship matrices results in a model for genetic evaluation where both pedigree and marker genotypes are used simultaneously for genetic evaluation, i.e. a single-step method for genomic evaluation. This paper provides the models and mathematical formulas, but a numerical implementation is needed before the methods are ready for use in practice. Such methods make it possible to incorporate phenotypes and genotypes on crossbreds into an existing genetic evaluation system, assuming that such a system is based on a single-step method.

The models for three-way crossbreeding investigated in this paper were four-variate models where each variable was measured in a specific population, $\mathcal {A}$, $\mathcal {B}$, $\mathcal {C}$ or $\mathcal {C}(\mathcal {A}\mathcal {B})$. The main scenario that we have in mind is a scenario where the four variables represent the same biological trait measured in four different genetic backgrounds and possibly different environments, but in principle the four variables could also be different biological traits. An extension of the model to a situation where multiple biological traits are measured in each of the four populations is in principle straightforward since the additive relationship matrices are the same, although in practice it may require the estimation of a very large number of genetic parameters. Extending the approaches to other types of models that are implemented in standard animal breeding software, like threshold models, models with indirect genetic effects, models for test-day records, etc. is also in principle straightforward. Finally, modifying the models to other scenarios with data recording, for example with records on $\mathcal {A}\mathcal {B}$ individuals or no records on one of the pure breeds, is also straightforward. In general, designing data recording for these complicated models is an issue, and for example to obtain precise estimates of the genetic correlation parameters, it would be important that the relationships between crossbred animals with records and purebred animals with records are close.

Two types of approaches for constructing additive relationships were presented, based on different assumptions about allele substitution effects of causal loci or SNPs. In the partial genetic approach, allele substitution effects of SNPs were assumed independent between breeds, whereas in the common genetic approach, they were assumed to be the same in different breeds. The partial genetic approach requires that alleles are traced according to breed of origin, which is feasible in some scenarios but may be difficult with sufficient accuracy in others. In particular, when crossbred $\mathcal {C}(\mathcal {A}\mathcal {B})$ animals are genotyped, a reasonable requirement is that breed $\mathcal {C}$ fathers are also genotyped which would make the tracing of the breed $\mathcal {C}$ paternal allele feasible, but the tracing of the breed of origin ($\mathcal {A}$ or $\mathcal {B}$) of the maternal allele may be more uncertain and depend on whether $\mathcal {A}\mathcal {B}$ mothers are genotyped (may not be due to logistical issues), maternal grandfathers are genotyped and maternal grandmothers are genotyped (may be difficult to obtain if these are from multiplier herds). An advantage of the common genetic approach is that the marker-based relationship matrix is easier to construct because tracing the breed of origin of alleles is not required, but a disadvantage may be the computational burden of using a larger relationship matrix. In addition, parameters in matrix $\varvec{\Gamma }$ need to be estimated and the sensitivity of genetic evaluation to these estimates is unknown. Future research using simulated and real data is needed to clarify the differences between the two approaches.

Other terminal crossbreeding systems are of interest in pig production. Models for two-way crossbreeding are relevant for sow-traits measured on animals from breed $\mathcal {A}$ and $\mathcal {B}$ and cross $\mathcal {A}\mathcal {B}$, and such models were presented in Christensen et al. [11] using partial genetic relationship matrices. An alternative to this partial genetic approach would be to use the common genetic approach presented here. The four-way crossbreeding system where crossbred $\mathcal {C}\mathcal {D}$ sires are mated to $\mathcal {A}\mathcal {B}$ dams to produce $(\mathcal {C}\mathcal {D})(\mathcal {A}\mathcal {B})$ pigs for slaughter, is also used in pig production. The approaches in this paper can be extended to such a system, and the resulting model would be a five-variate model. Using the partial genetic approach, there would be four breed-specific partial relationship matrices and two breed-segregation partial relationship matrices, and the corresponding model for purebred and crossbred performances would contain 14 genetic parameters, whereas using the common genetic approach, the model for purebred and crossbred performances would contain 15 genetic parameters.

Many papers have reported genetic correlations between purebred and crossbred performances [21–26]. The reported estimated correlations ranged from 0.38 to 0.946, depending on trait and on differences in the environment, and in general with relatively high standard error on the estimates. The higher the genetic correlation, the less gain there will be by including crossbred data into the genetic evaluation system. All these results are from two-way crosses, and the authors are not aware of publications based on data from three-way crossbreeding where data in purebred and crossbred populations are considered to be different traits. The models presented in this paper should be useful to investigate such data from three-way crossbreeding.

## Conclusion

Models for genetic evaluation in the three-way crossbreeding system are presented. These models provide estimated breeding values for both purebred and crossbred performances, and can use pedigree-based or marker-based relationships, or combined relationships based on both pedigree and marker information. This provides a framework that allows information from three-way crossbred animals to be incorporated into a genetic evaluation system.

## Appendix

Here, explicit and detailed derivations of the additive relationships across purebred and crossbred animals related to the $\mathcal {C}(\mathcal {A}\mathcal {B})$ crossbreeding system are presented.

In the derivation, both partial genetic and common genetic approaches for the variance-covariance of genetic effects for crossbred $\mathcal {C}(\mathcal {A}\mathcal {B})$ performance are inspired by the derivation in Lo et al. [13] of formulas (3) and (4). Lo et al. [13] based their derivation on the genotypic value expressed as a sum over loci of effects of paternal and maternal alleles:

$$\begin{aligned} g_i = \mu + \sum _j (\alpha _{s_i^j} + \alpha _{d_i^j}) , \end{aligned}$$

where $\alpha _{s_i^j}$ and $\alpha _{d_i^j}$ are the additive effects of the paternal and maternal alleles, respectively, and these effects depend on the breed of origin *b* of the alleles *j*, such that ${\text {E}}[\alpha _{s_i^j}\mid s_i\in b]=\epsilon _j^b$ and ${\text {E}}[\alpha _{d_i^j}\mid d_i\in b]=\epsilon _j^b$, where the expectation is taken across all individuals in breed *b* and symbol $\in$ is used to denote breed of origin of an allele. The term $\epsilon _j^b$ is the mean additive effect and this is different between breeds due to different allele frequencies in different breeds. To be explicit, $\epsilon ^b_{j}= p^b_j a_j + (1-p^b_j)(-a_j) = (p^b_j-1/2)2a_j$ where $p^b_j$ is the allele frequency in breed *b* and $a_j$ is the additive effect of the *j*-th allele. Above, both expectations and allele frequencies refer to the base populations, and it is assumed that in each base population, alleles are assigned randomly to individuals. It is further assumed that effects for different loci are independent. Here, we introduce the notation $\alpha ^b_{s_i^j}=\alpha _{s_i^j}-\epsilon _j^b$ when $s^j_i\in b$ and $\alpha ^b_{d_i^j}=\alpha _{d_i^j}-\epsilon _j^b$ when $d^j_i\in b$, such that the expectations of the $\alpha ^b$s are equal to 0.

First, pedigree-based additive genetic relationships are derived using the partial genetic approach and common genetic approach, respectively, and second the corresponding marker-based relationships are derived.

### Additive genetic model for crossbred $\mathcal {C}(\mathcal {A}\mathcal {B})$ performance: partial genetic approach

Contrary to Lo et al. [13] and Garcia-Cortes and Toro [14], in the derivation presented here, we first split the genotypic values according to breeds of origin instead of computing the variances and covariances and then splitting them. The reason for this is for similarity with the derivation of the corresponding genomic model using the partial genetic approach that appears in a following subsection.

For the $\mathcal {C}(\mathcal {A}\mathcal {B})$ crossbred animals studied here, the paternal allele is always breed $\mathcal {C}$ and the maternal allele is either breed $\mathcal {A}$ or $\mathcal {B}$ with equal probability, and therefore the genotypic value becomes:

$$\begin{aligned} g_i&= \mu + \sum _j \alpha _{s_i^j} + \sum _{j: d^j_i \in \mathcal {A}} \alpha _{d_i^j} + \sum _{j: d^j_i \in \mathcal {B}} \alpha _{d_i^j} \\&= \tilde{\mu }_{\mathcal {C}(\mathcal {A}\mathcal {B})} + \sum _j \alpha ^{\mathcal {C}}_{s_i^j} + \sum _{j: d^j_i \in \mathcal {A}} \alpha ^{\mathcal {A}}_{d_i^j} + \sum _{j: d^j_i \in \mathcal {B}} \alpha ^{\mathcal {B}}_{d_i^j} \\&\quad + 0.5\sum _j r_{d^j_i}(\epsilon _j^{\mathcal {A}}-\epsilon _j^{\mathcal {B}}) \\&= \tilde{\mu }_{\mathcal {C}(\mathcal {A}\mathcal {B})} + g^{\mathcal {C}}_{\mathcal {C}(\mathcal {A}\mathcal {B}),i} + g^{\mathcal {A}}_{\mathcal {C}(\mathcal {A}\mathcal {B}),i} + g^{\mathcal {B}}_{\mathcal {C}(\mathcal {A}\mathcal {B}),i} + g^{\mathcal {A}\mathcal {B}}_{\mathcal {C}(\mathcal {A}\mathcal {B}),i}, \end{aligned}$$

where $\tilde{\mu }_{\mathcal {C}(\mathcal {A}\mathcal {B})} = \mu + \sum _j\epsilon _j^{\mathcal {C}} + 0.5 (\sum _j\epsilon _j^{\mathcal {A}}+\sum _j\epsilon _j^{\mathcal {B}})$, $r_{d^j_i}=1$ when $d^j_i\in \mathcal {A}$ and $r_{d^j_i}=-1$ when $d^j_i\in \mathcal {B}$, and terms $\mathbf {g}^{\mathcal {C}}_{\mathcal {C}(\mathcal {A}\mathcal {B})}$, $\mathbf {g}^{\mathcal {A}}_{\mathcal {C}(\mathcal {A}\mathcal {B})}$, $\mathbf {g}^{\mathcal {B}}_{\mathcal {C}(\mathcal {A}\mathcal {B})}$ and $\mathbf {g}^{\mathcal {A}\mathcal {B}}_{\mathcal {C}(\mathcal {A}\mathcal {B})}$ are defined implicitly. In this way, the genotypic value has been split into partial genetic effects, where the terms $g^{\mathcal {C}}_{\mathcal {C}(\mathcal {A}\mathcal {B})}$, $\mathbf {g}^{\mathcal {A}}_{\mathcal {C}(\mathcal {A}\mathcal {B})}$, $\mathbf {g}^{\mathcal {B}}_{\mathcal {C}(\mathcal {A}\mathcal {B})}$ are breed of origin specific and $\mathbf {g}^{\mathcal {A}\mathcal {B}}_{\mathcal {C}(\mathcal {A}\mathcal {B})}$ is a breed-segregation term.

For the $\mathcal {A}\mathcal {B}$ crossbred sows (where for simplicity of notation it is assumed that their fathers are breed $\mathcal {A}$ and mothers breed $\mathcal {B}$),

$$\begin{aligned} g_i&= \mu + \sum _j \alpha _{s_i^j} + \sum _j \alpha _{d_i^j} = \tilde{\mu }_{\mathcal {A}\mathcal {B}} + \sum _j \alpha ^{\mathcal {A}}_{s_i^j} + \sum _j \alpha ^{\mathcal {B}}_{d_i^j} \\ &=\,\tilde{\mu }_{\mathcal {A}\mathcal {B}} + g^{\mathcal {A}}_{\mathcal {A}\mathcal {B},i} + g^{\mathcal {B}}_{\mathcal {A}\mathcal {B},i}, \end{aligned}$$

with $\tilde{\mu }_{\mathcal {A}\mathcal {B}} = \mu + \sum _j\epsilon _j^{\mathcal {A}}+\sum _j\epsilon _j^{\mathcal {B}}$, and terms $\mathbf {g}^{\mathcal {A}}_{\mathcal {A}\mathcal {B}}$ and $\mathbf {g}^{\mathcal {B}}_{\mathcal {A}\mathcal {B}}$ defined implicitly.

For a purebred animal of breed *b*

$$\begin{aligned} g_i&= \mu + \sum _j \alpha _{s_i^j} + \sum _j \alpha _{d_i^j} = \tilde{\mu }_{b} + \sum _j \alpha ^{b}_{s_i^j} + \sum _j \alpha ^{b}_{d_i^j} \\&= \tilde{\mu }_{b} + g_{b,i}, \end{aligned}$$

where $\tilde{\mu }_{b} = \mu + 2\sum _j\epsilon _j^{b}$ and $g_{b,i}=\sum _j \alpha ^{b}_{s_i^j} + \sum _j \alpha ^{b}_{d_i^j}$ is the breeding value for crossbred $\mathcal {C}(\mathcal {A}\mathcal {B})$ performance. From this, the three vectors of breeding values for crossbred $\mathcal {C}(\mathcal {A}\mathcal {B})$ performance, $\mathbf {g}_{\mathcal {A}}$, $\mathbf {g}_{\mathcal {B}}$ and $\mathbf {g}_{\mathcal {C}}$, are defined.

In this way, a breed-specific partial genetic effect has been defined for all animals containing the specific breed, and a breed-segregation partial genetic effect has been defined for crossbred $\mathcal {C}(\mathcal {A}\mathcal {B})$ animals. The resulting variance-covariance matrices are as shown in the “Methods” section.

Note that the different means $\tilde{\mu }_{\mathcal {C}(\mathcal {A}\mathcal {B})}$, $\tilde{\mu }_{\mathcal {A}\mathcal {B}}$, $\tilde{\mu }_{\mathcal {A}}$, $\tilde{\mu }_{\mathcal {B}}$ and $\tilde{\mu }_{\mathcal {C}}$ should strictly speaking be included into the genetic effects and breeding values, but they have been omitted here, since the genetic values are for performance in a specific $\mathcal {C}(\mathcal {A}\mathcal {B})$ cross and these means cannot be inferred from data.

The variance of the breed-segregation term for a $\mathcal {C}(\mathcal {A}\mathcal {B})$ animal equals

$$\begin{aligned} Var(g^{\mathcal {A}\mathcal {B}}_{\mathcal {C}(\mathcal {A}\mathcal {B}),i}) &= {\text {Var}}(0.5\sum _j r_{d^j_i}(\epsilon _j^{\mathcal {A}}-\epsilon _j^{\mathcal {B}})) \\ &= \sum _j(\epsilon _j^{\mathcal {A}}-\epsilon _j^{\mathcal {B}})^2/4= \sigma _{g,\mathcal {A}\mathcal {B}}^2/2 , \end{aligned}$$

where $\sigma _{g,\mathcal {A}\mathcal {B}}^2=\sum _j(\epsilon ^{\mathcal {A}}_j-\epsilon ^{\mathcal {B}}_j)^2/2$ is the breed-segregation variance, i.e. the additional genetic variance in an F2 cross compared to an F1 cross. The assumption that has been used here is that the $\epsilon ^{\mathcal {A}}_j$s and $\epsilon ^{\mathcal {B}}_j$s are fixed constants.

### Additive genetic model for crossbred $\mathcal {C}(\mathcal {A}\mathcal {B})$ performance: common genetic approach

Using the notation defined above, the genetic value for crossbred performance of purebred *b* animal *i* equals

$$\begin{aligned} g_i = \mu + \sum _{j=1}^N \alpha _{s_i^j} + \sum _{j=1}^N \alpha _{d_i^j} = \mu + \sum _{j=1}^N (\alpha ^b_{s_i^j} + \alpha ^b_{d_i^j} + 2 \epsilon ^b_{j}), \end{aligned}$$

where the $\alpha ^b_{s^j_i}$s and $\alpha ^b_{d^j_i}$s are independent between breeds, and *N* is the number of genes. Compared to the partial genetics approach, here we assume that both the $\alpha ^b$s and the $\epsilon ^b_{j}$s are random variables. The randomness in the $\alpha ^b$s is because different animals inherit different alleles, but the randomness in $\epsilon ^b_{j}$ has a different origin. Note that the differences in $\epsilon ^b_{j}$ between breeds is due to differences in allele frequencies, and as explained previously, $\epsilon ^b_{j}= p^b_j a_j + (1-p^b_j)(-a_j) = (p^b_j-1/2)2a_j$, where $p^b_j$ is the allele frequency and $a_j$ is the allelic effect. Assigning prior distributions with expectations 1/2 to the allele frequencies as in Christensen [20] corresponds to assigning a prior distribution with expectation 0 and variance proportional to $(a_j)^2$ to $\epsilon ^b_{j}$ for all loci. Furthermore, assuming that prior distributions for allele frequencies are correlated between breeds implies that covariances of the $\epsilon ^b_{j}$s become proportional to $(a_j)^2$.

Thus, we assume that the $\epsilon ^b_{j}$s are random variables that are independent of the $\alpha ^b_{s^j_i}$s and $\alpha ^b_{d^j_i}$s and have a mean of 0. Furthermore, we assume that the $\epsilon ^b_{j}$s are correlated between breeds,

$$\begin{aligned} {\text {Var}}\left[ \begin{array}{c} \epsilon ^{\mathcal {A}}_{j} \\ \epsilon ^{\mathcal {B}}_{j} \\ \epsilon ^{\mathcal {C}}_{j} \\ \end{array} \right] = \left[ \begin{array}{ccc} \tau ^2_{\epsilon ,\mathcal {A}} &{} \tau _{\epsilon ,\mathcal {A},\mathcal {B}} &{} \tau _{\epsilon ,\mathcal {A}, \mathcal {C}} \\ \tau _{\epsilon ,\mathcal {A},\mathcal {B}} &{} \tau ^2_{\epsilon ,\mathcal {B}} &{} \tau _{\epsilon ,\mathcal {A},\mathcal {C}} \\ \tau _{\epsilon ,\mathcal {A},\mathcal {C}} &{} \tau _{\epsilon ,\mathcal {A},\mathcal {B}} &{} \tau ^2_{\epsilon ,\mathcal {C}} \\ \end{array} \right] (a_j)^2. \end{aligned}$$

The variable $\alpha ^b_{s_i^j}$ when $s^j_i\in b$ is a random variable with mean 0, and since it may be expressed as $\alpha ^b_{s_i^j}=(z_{s_i^j} -p^b_j)a_j$ where $z_{s_i^j}=0, 1$ for paternal allele being 1 and 2, respectively, we see that the variance of $\alpha ^b_{s_i^j}$ is proportional to $(a_j)^2$. Similarly, $\alpha ^b_{d_i^j}$ is a random variable with mean 0 and variance proportional to $(a_j)^2$. For animals in the base population of the pedigree, the variances of $\alpha ^b_{s_i^j}$ and $\alpha ^b_{d_i^j}$ equal $\tau ^2_{\alpha , b}(a_j)^2$ with $\tau ^2_{\alpha , b}={\text {E}}[{\text {Var}}[(z_{s_i^j} -p^b_j)\mid p_j^b]]={\text {E}}[p^b_j(1-p^b_j)]={\text {E}}[p^b_j] -({\text {Var}}[p^b_j]+{\text {E}}[p^b_j]^2)=1/4-\tau ^2_{\epsilon , b}$ where expectation is taken with respect to the prior distribution of $p^b_j$, and the mutual covariances between $\alpha ^b_{s_i^j}$, $\alpha ^b_{s_{i^{\prime }}^j}$, $\alpha ^b_{d_i^j}$ and $\alpha ^b_{d_{i^{\prime }}^j}$ are all zero when $i\ne i^{\prime }$.

Therefore, in the base population of the pedigree, the variances and covariances of the genetic values $g_i=\sum _j(\alpha ^b_{s_i^j}+\alpha ^b_{d_i^j}+2\epsilon ^b_j)$ become

$$\begin{aligned} {\text {Var}}(g_i) = (2\tau ^2_{\alpha , b}+ 4\tau ^2_{\epsilon , b}) \sum _j (a_j)^2 , \end{aligned}$$

for an individual in breed *b*,

$$\begin{aligned} {\text {Cov}}(g_i,g_{i^{\prime }})= 4\tau ^2_{\epsilon , b}\sum _j (a_j)^2, \end{aligned}$$

for two different individuals in breed *b*, and finally

$$\begin{aligned} {\text {Cov}}(g_i,g_{i^{\prime }})= 4\tau _{\epsilon , b,b^{\prime }}\sum _j(a_j)^2, \end{aligned}$$

for two individuals in different breeds *b*, $b^{\prime }$.

Defining new parameters $\sigma _g^2= \sum _j (a_j)^2(2\tau ^2_{\alpha , b}+ 2\tau ^2_{\epsilon , b})$ and $\gamma _b=4 \tau ^2_{\epsilon , b}/(2\tau ^2_{\alpha , b}+ 2\tau ^2_{\epsilon , b})$, we see that the elements of the variance-covariance matrix within breed is defined by:

$$\begin{aligned} {\text {Var}}(g_i)= \sigma _g^2(1 + \gamma _b/2), \end{aligned}$$

for an individual in breed *b*, and

$$\begin{aligned} {\text {Cov}}(g_i,g_{i^{\prime }})= \sigma _g^2\gamma _b, \end{aligned}$$

for two individuals in breed *b*, i.e. animals in the base population are inbred with coefficient $\gamma _b/2$ and related with relationship coefficient $\gamma _b$. Note that from $\tau ^2_{\alpha , b}+ \tau ^2_{\epsilon , b}=1/4$, we see that $\sigma _g^2= \sum _j (a_j)^2/2$ does not depend on breed and $\gamma _b=8 \tau ^2_{\epsilon , b}$. Furthermore, define $\gamma _{b,b^{\prime }}=8\tau _{\epsilon , b,b^{\prime }}$, then

$$\begin{aligned} {\text {Cov}}(g_i,g_{i^{\prime }})= \sigma _g^2 \gamma _{b,b^{\prime }}, \end{aligned}$$

for two individuals in different breeds *b*, $b^{\prime }$, i.e. base animals in different breeds are related. Therefore, a joint relationship matrix is specified among all base animals, and by applying the usual recursive definition, an additive relationship matrix is defined across all animals as shown in the “Methods” section.

Note that the breed-segregation term disappears here, since the differences in the $\epsilon ^{\mathcal {A}}$ and $\epsilon ^{\mathcal {B}}$ terms are incorporated into the $\varvec{\Gamma }$ matrix. However, breed-segregation variance is still present under this model, which is illustrated by

$$\begin{aligned} {\text {Var}}(g^{\mathcal {A}\mathcal {B}}_{\mathcal {C}(\mathcal {A}\mathcal {B}),i})&= {\text {Var}}(0.5\sum _j r_{d^j_i}(\epsilon _j^{\mathcal {A}}-\epsilon _j^{\mathcal {B}})) \\&= \sum _{j=1}^n {\text {E}}((\epsilon _j^{\mathcal {A}}-\epsilon _j^{\mathcal {B}})^2)/4 \\&= (\tau ^2_{\epsilon ,\mathcal {A}}+\tau ^2_{\epsilon ,\mathcal {B}} -2\tau _{\epsilon ,\mathcal {A},\mathcal {B}}) \sum _j (a_j)^2/4\\&= (\gamma _{\mathcal {A}} + \gamma _{\mathcal {B}}-2\gamma _{\mathcal {A},\mathcal {B}})\sigma ^2_g/16 \\&= \sigma _{g,\mathcal {A}\mathcal {B}}^2/2 , \end{aligned}$$

where $\sigma _{g,\mathcal {A}\mathcal {B}}^2= \sigma ^2_g((\gamma _{\mathcal {A}}+\gamma _{\mathcal {B}})/2-\gamma _{\mathcal {A},\mathcal {B}})/4$ is the breed-segregation variance, i.e. the additional genetic variance in an F2 cross compared to an F1 cross; see Legarra et al. (2015) [15].

### Genomic model for crossbred $\mathcal {C}(\mathcal {A}\mathcal {B})$ performance: partial genetic approach

Based on pedigree relationships, the randomness comes from not knowing which alleles are inherited, but when having marker genotypes this is actually known. Here, the randomness comes from assigning distributions on effects. In addition, causal loci are replaced by SNPs that are in linkage disequilibrium with causal loci, but for shortness of notation we will use the same notation for the effects of SNPs as that used for the causal loci.

Now, for SNP *j*

$$\begin{aligned} \alpha ^b_{s_i^j}=(z_{s_i^j}-p^b_j)\beta ^b_j, \ \ \alpha ^b_{d_i^j}=(z_{d_i^j}-p^b_j)\beta ^b_j, \end{aligned}$$

where $z_{s_i^j}=0, 1$ for paternal allele being 1 and 2, respectively, $z_{s_i^j}$ is defined similarly for the maternal allele, $p^b_j$ denotes the allele frequency of breed *b* base population, and $\beta ^b_j$ is the breed *b* specific allele substitution effect which is assumed to be Gaussian distributed with mean 0. Breed-specific partial genetic effects are therefore as follows:

$$\begin{aligned} g_{\mathcal {A},i}&= \sum _j (z_{s_i^j}+z_{d_i^j}-2p^{\mathcal {A}}_j)\beta ^{\mathcal {A}}_j , \\ g^{\mathcal {A}}_{\mathcal {A}\mathcal {B},i}&= \sum _j (z_{s_i^j}-p^{\mathcal {A}}_j)\beta ^{\mathcal {A}}_j , \\ g^{\mathcal {A}}_{\mathcal {C}(\mathcal {A}\mathcal {B}),i}&= \sum _{j: d^j_i \in \mathcal {A}} (z_{d_i^j}-p^{\mathcal {A}}_j)\beta ^{\mathcal {A}}_j , \end{aligned}$$

for breed $$\mathcal {A}$$,

$$\begin{aligned} g_{\mathcal {B},i}&= \sum _j (z_{s_i^j}+z_{d_i^j}-2p^{\mathcal {B}}_j)\beta ^{\mathcal {B}}_j , \\ g^{\mathcal {B}}_{\mathcal {A}\mathcal {B},i}&= \sum _j (z_{d_i^j}-p^{\mathcal {B}}_j)\beta ^{\mathcal {B}}_j , \\ g^{\mathcal {B}}_{\mathcal {C}(\mathcal {A}\mathcal {B}),i}&= \sum _{j: d^j_i \in \mathcal {B}} (z_{d_i^j}-p^{\mathcal {B}}_j)\beta ^{\mathcal {B}}_j , \end{aligned}$$

for breed $\mathcal {B}$, and

$$\begin{aligned} g_{\mathcal {C},i}&= \sum _j (z_{s_i^j}+z_{d_i^j}-2p^{\mathcal {C}}_j)\beta ^{\mathcal {C}}_j , \\ g^{\mathcal {C}}_{\mathcal {C}(\mathcal {A}\mathcal {B}),i}&= \sum _j (z_{s_i^j}-p^{\mathcal {C}}_j)\beta ^{\mathcal {C}}_j , \end{aligned}$$

for breed $\mathcal {C}$. The breed-segregation partial genetic effect is $g^{\mathcal {A}\mathcal {B}}_{\mathcal {C}(\mathcal {A}\mathcal {B}),i}= 0.5\sum _j r_{d^j_i}(\epsilon _j^{\mathcal {A}}-\epsilon _j^{\mathcal {B}})$ where $r_{d^j_i}=1$ when $d^j_i\in \mathcal {A}$ and $r_{d^j_i}=-1$ when $d^j_i\in \mathcal {B}$ and $\epsilon _j^{\mathcal {A}}-\epsilon _j^{\mathcal {B}}=p^{\mathcal {A}}_j\beta ^{\mathcal {A}}_j - p^{\mathcal {B}}_j\beta ^{\mathcal {B}}_j$.

An equivalent formulation of the model is to use marker-based partial relationship matrices instead of breed-specific allele substitution effects. Define breed *b* specific allele content matrices as matrix $\mathbf {m}^b$ with entries $m^b_{ij}=z_{s_i^j}+z_{d_i^j}$ for purebred *b* animals, matrices $\mathbf {z}^{\mathcal {A}}$ and $\mathbf {z}^{\mathcal {B}}$ with entries $z_{s_i^j}$ and $z_{d_i^j}$, respectively, for crossbred $\mathcal {A}\mathcal {B}$ animals, matrix $\mathbf {z}^{\mathcal {C}}$ with entries $z_{s_i^j}$ for crossbred $\mathcal {C}(\mathcal {A}\mathcal {B})$ animals, and finally matrices $\mathbf {z}_p^{\mathcal {A}}$ and $\mathbf {z}_p^{\mathcal {B}}$ with entries $z_{d_i^j}$ and $z_{d_i^j}$, respectively, for crossbred $\mathcal {C}(\mathcal {A}\mathcal {B})$ animals when the breed-specific allele is inherited and zero otherwise. From these breed-specific allele content matrices, marker-based breed-specific partial relationship matrices are constructed and these are as in the “Methods” section. Note that the scalings of these matrices are $s^b={\text {Var}}(\beta ^b_j)/\sigma ^2_b$ for $b=\mathcal {A},\mathcal {B},\mathcal {C}$. The breed-segregation partial relationship matrix is defined as $G^{\mathcal {A}\mathcal {B}}_{i,i^{\prime }} = \sum _j r_{d^j_i} r_{d^j_{i^{\prime }}}/(2n)$.

Independence between vectors, $\mathbf {g}^{(\mathcal {A})}$, $\mathbf {g}^{(\mathcal {B})}$, $\mathbf {g}^{(\mathcal {C})}$ and $\mathbf {g}^{\mathcal {A}\mathcal {B}}_{\mathcal {C}(\mathcal {A}\mathcal {B})}$ requires that allele substitution effects $\beta _j^{\mathcal {A}}$, $\beta _j^{\mathcal {B}}$ and $\beta _j^{\mathcal {C}}$ are independent between breeds, and also that they are independent of the $\epsilon ^{\mathcal {A}}_j-\epsilon ^{\mathcal {B}}_j$. First, independence between $\beta _j^{\mathcal {A}}$, $\beta _j^{\mathcal {B}}$ and $\beta _j^{\mathcal {C}}$ seems to somehow contradict the assumption of additive gene effect $a_j$ being the same independent of breed of origin, but may be justified if there is no persistence of the phase between markers and QTL in different breeds. Second, $\epsilon ^{\mathcal {A}}_j-\epsilon ^{\mathcal {B}}_j=p^{\mathcal {A}}_j\beta ^{\mathcal {A}}_j - p^{\mathcal {B}}_j\beta ^{\mathcal {B}}_j$ being independent of $\beta _j^{\mathcal {A}}$ and $\beta _j^{\mathcal {B}}$ cannot strictly hold when $\beta _j^{\mathcal {A}}$ and $\beta _j^{\mathcal {B}}$ are also independent. However, as explained by de los Campos et al. [27] the fact that multiple markers are likely to track the same QTL questions the assumptions of additivity and independence of SNP allele substitution effects between loci, and therefore the genomic model is a rough approximation to the reality of the additive model of causal effects.

### Genomic model for crossbred $\mathcal {C}(\mathcal {A}\mathcal {B})$ performance: common genetic approach

A genomic version of the model with common genetic effects across breeds can be formulated by replacing $\alpha ^b_{s_i^j}$ by $(z_{s_i^j}-p^b_j)\beta _j$, $\alpha ^b_{d_i^j}$ by $(z_{d_i^j}-p^b_j)\beta _j$, and $\epsilon ^b_j$ by $(p^b_j-1/2)\beta _j$ where 1/2 is the common allele frequency and $\beta _j$ is the allele substitution effect. Then,

$$\begin{aligned} \alpha ^b_{s_i^j}+\epsilon ^b_j=(z_{s_i^j}-0.5)\beta _j, \ \ \alpha ^b_{d_i^j}+\epsilon ^b_j=(z_{d_i^j}-0.5)\beta _j . \end{aligned}$$

The resulting model becomes:

$$\begin{aligned} g_i&= \mu + \sum _j (z_{s_i^j}-0.5)\beta _j + (z_{d_i^j}-0.5)\beta _j \\&= \mu + \sum _j (m_{i,j}-1)\beta _j . \end{aligned}$$

The marker-based relationship matrix is therefore constructed as usual across all genotyped animals:

$$\begin{aligned} \mathbf {G}=\frac{(\mathbf {m}-\mathbf {1}\mathbf {1}^\mathrm{{\scriptstyle {T}}})(\mathbf {m}-\mathbf {1}\mathbf {1}^\mathrm{{\scriptstyle {T}}})^\mathrm{{\scriptstyle {T}}}}{s} , \end{aligned}$$

where *s* is scaling parameter.
